# Fine Endmesolithic fish caviar meal discovered by proteomics in foodcrusts from archaeological site Friesack 4 (Brandenburg, Germany)

**DOI:** 10.1371/journal.pone.0206483

**Published:** 2018-11-28

**Authors:** Anna Shevchenko, Andrea Schuhmann, Henrik Thomas, Günter Wetzel

**Affiliations:** 1 Max Planck Institute of Molecular Cell Biology and Genetics (MPI-CBG), Dresden, Germany; 2 Brandenburgisches Landesamt für Denkmalpflege und Archaeologisches Landesmuseum (BLDAM), Aussenstelle Cottbus, Germany; Max Planck Institute for the Science of Human History, GERMANY

## Abstract

The role of aquatic resources in ancient economies and paleodiet is important for understanding the evolution of prehistorical societies. Charred food remains from ancient pottery are valuable molecular evidence of dietary habits in antiquity. However, conventional archaeometric approaches applied in their analysis lack organismal specificity, are affected by abundant environmental contaminants, do not elucidate food processing recipes and are limited in the inland regions where diverse dietary resources are available. We performed proteomics analysis of charred organic deposits adhered on early ceramics from Mesolithic-Neolithic inland site Friesack 4 (Brandenburg, Germany). One of pots—a small coarse bowl radiocarbon dated to the end of the 5^th^ millennium BC—was attributed to Endmesolithic pottery. Proteomics of foodcrust from this vessel identified fine carp roe meal and revealed details of a prehistorical culinary recipe. Ancient proteins were unequivocally distinguished from contemporary contaminants by computing deamidation ratios of glutamine residues. These data paint a broader picture of the site-specific exploitation of aquatic resources and contribute to better understanding of the dietary context of Neolithic transition in European inland.

## Introduction

The major inducements underlying developments in prehistorical societies were access and use of natural resources, which also played a key role in Neolithic transition across Europe. The role of aquatic resources during this critical phase in European prehistory remains in the focus of longstanding debates particularly regarding inland regions [1–6].

Inland prehistorical localities were often situated close to rivers or lakeshores. Although it is conceivable that fish and water plants were a part of the diet of their inhabitants their general subsistence strategies did not necessarily rely on freshwater resources [7]. Our understanding of the role of aquatic resources in economy of prehistorical aceramic communities relies on assemblage of recovered artefacts: zooarchaeological materials (shellfish, bones, scales), catching or processing tools and related art objects—zoomorphic figurines, paintings, adornments. Additionally, analysis of DNA recovered from ancient fishbones was used for the identification of fish species [8]. Systematic carbon and nitrogen isotopic analysis of associated human remains (bones, teeth or hairs) shed light on dietary importance of aquatic products [9, 10]. However, the related archaeological artefacts are absent at many sites. Fish bones and scales are not well preserved in acidic soils and are often underrepresented in archaeofaunal assemblages if no environmental sampling or sieving was applied. Isotopic composition of human remains is influenced by many factors including physiology of the deceased individual [11, 12]. Apart of it, analysis of stable isotopes is significantly limited in the regions where diverse dietary resources are available and is not applicable for estimating of dietary contribution of fresh water products at inland sites [7].

Neolithic transition in European inland was accompanied by appearance and spread of early ceramics which serialization is important for chronological reconstruction. Charred food remains (or foodcrusts) often found on these early pottery is a valuable molecular evidence, particularly for the evaluation of dietary role of aquatic resources in prehistorical communities. Isotopic profile and composition of small organic molecules in ceramic foodcrusts—lipids, fatty acids, sterols and a few known molecular biomarkers,—enable distinguishing terrestrial, marine and fresh water materials (reviewed in [13, 14]). However, they do not identify the source animal species and reveal little details about the cooking recipes. Also employed analytical procedures are sensitive to contemporary contaminants.

Analysis of proteins in archaeological organic residues is advantageous for the interpretation of their complex composition (reviewed in [15]) and are essential also for ceramic foodcrusts. Ancient proteins carry aging-specific chemical modification which helps distinguishing them from modern proteinous contaminants [16–18]. Protein sequences provide direct information about the species of origin [19–21]. Furthermore, their biological properties (enzymatic activity or organismal localization) could elucidate how raw materials were processed [22, 23]. Thus, the identification of protein composition in foodcrusts might also assist in reconstruction of cooking recipe of prehistorical foods.

In this work we present proteomics analysis of foodcrusts from ceramics recovered at the inland Mesolithic-Neolithic site Friesack 4 (Brandenburg, Germany). The site is known for its complex stratigraphy and excellent preservation of numerous predominantly Mesolithic artefacts in the over 2m deep cultural occupation layer. This unique collection also comprises early ceramics including Friesack-Boberger group [24, 25] which represents the most southern example of Mesolithic pottery in the Northern European lowland. A small fraction of the pottery fragments from Friesack preserves visible foodcrusts. Their protein compositions shed light on prehistorical cooking practice, paleodiet and help understanding the process of transformation from hunter-gatherer groups to sedentary society in European inland.

## Material and methods

### Geographical location and history of exploitation of Friesack 4

**Fig 1:**
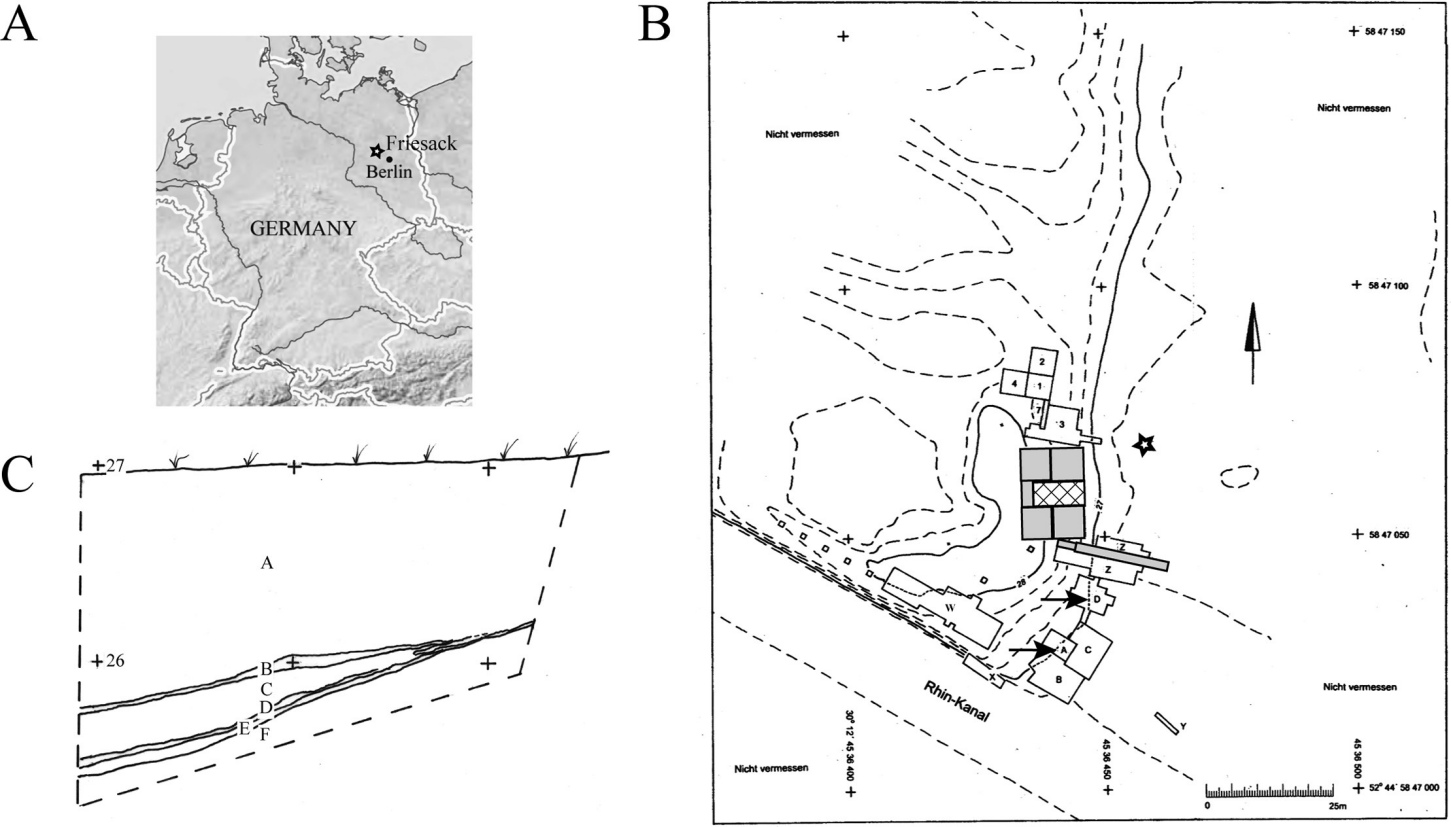
Archaeological site Friesack 4. Panel A: Location in Havelland administrative district, Brandenburg, Germany. Panel B: Layout of archaeological excavations undertaken at the Friesack 4 site: shaded area–leaded by M. Schneider (1916–1928); filled grey areas—H. Reinerth (R 1-5/1940); designated with “W”—S. Wenzel (2000–2001); designated with 1–4, 7, A-D, X-Z—B. Gramsch (1977–1989; 1998). The position of the test trench 1979 is designated with asterisk; quadrats A and D are pointed with arrows (graphic rendering the original map from Bodo Hildebrand (†) and Dieter Becker). Panel C. South profile of the test trench 1979: A—peat; B, D–yellowish grey light sand; C, E–organic silt; F–Pleistocene sand (graphic rendering the original drawing from B. Gramsch).

Friesack 4 is a Mesolithic-Neolithic bog site near a small town Friesack (52° 43′ 59″ N, 12° 34′ 59″ E) in Havelland administrative district (Brandenburg, Germany) ca 70km west-northwest from Berlin (Fig 1). It was discovered by amateur archaeologist Max Schneider at the beginning of the 20^th^ century when amelioration and draining bogs along Rhin River revealed first artefacts [26], and later explored by several expeditions leaded by H. Reinerth, S. Wenzel and particularly B. Gramsch (Fig 1).

Friesack 4 is situated in the Warsaw-Berlin ice margin valley on the shore of the Rhin River. Between Preboreal and Atlantic the landscape around the site presented afforested lowland along the water boundary interjected by ground moraines. According to archaeobotanical, palynological and zooarchaeological data, the region was first grown over with mixed pine and birch forest which was later replaced by deciduous trees, and was inhabited by broad variety of wild animals [27–30]. From as early as the end of the 10^th^ millennium BC groups of Mesolithic hunter-gatherers frequented at the Friesack site which was located at the shore of a lake [27]. Depositional events at the site suggested that till the end of Mesolithic in the middle of the 6^th^ Millennium BC it was visited 50–60 times [31]. Elevation of water level in terminal Boreal and Atlantic caused partial inundation and formation of massive fens around Friesack 4 and also probably resulted in a short occupational pause at the site [32]. Despite significantly changed landscape the territory remained attractive for hunting and fishing in terminal Mesolithic and was later occasionally visited by Neolithic groups settled in the Friesack vicinity [24]. The site was utilized exclusively as a seasonal hunting station occupied between early spring and late fall [33] rather than a year-around settlement. Trace amount of pollen detected at Friesack 4 [27] suggested that the place was not used as a cropland. Most likely Neolithic settlers cultivate the land south or west from the site.

### Friesack 4 collection of archaeological artefacts

Friesack 4 collection of artefacts comprises more than 150000 objects, the vast majority of which are Mesolithic. Apart of numerous lithics, it also includes objects made from organic materials: bone, wood, bast, pitch, antler—which have been well preserved in the natural environment of peat bog interspaced with sandy layers [28]. The collection encompasses nearly all groups of artefacts manufactured by Mesolithic occupants: from arrowheads and bast nets to decorated carapace of freshwater turtle and birch bark tar chewing gum–and shows substantial similarity with Maglemose culture [34, 35]. Ceramic fragments represent a very small fraction of the collection. No prehistorical burials were found in the closed vicinity apart of remains of two individuals briefly mentioned in the excavation reports from 40^th^, and a few single fragments reported at the site [28, 36].

Whereas the Mesolithic history of Friesack 4 has been intensively studied [28–30, 32, 35, 36] little is known about later phases of exploitation of the site. Only in a few recent years, its less abundant Neolithic artefacts including unique samples of early ceramics awakened interest of archaeologists [24, 25, 33].

### Archaeological samples

**#3258** (Inv. No 1977:7/3258 T): a group of 12 small to middle-size shards of the same fragment of a coarse undecorated pottery. The group was recovered from the lowest occupational layer (#4) of the south profile of a test trench made in the refuse zone northeast of the settlement by B.Gramsch in 1979 (Landesmuseum für Ur- und Frühgeschichte Potsdam, Germany) (Fig 1). The foodcrusts were adhered at inner surface of basal and rim shards. Individual shards were glued together shortly before sampling organic deposit. Radiocarbon dating, typology and reconstruction of the vessel profile are presented in this work.

**#3251.1** (Inv. No 1977:7/3251.1): a body shard of round-bodied vessel decorated with three drop-shaped imprints. It was recovered closely to the position of the #3258 from the layer #5 of the same trench in 1979 (Fig 1). Charred organic crust on the internal surface was radiocarbon dated 3946–3776 calBC (ARR 15048). The fragment was attributed to Early Neolith, Brzesc Kujawski group (?) ([24] Abb.6.6; [25] Abb.6.2).

**#3157.1** (Inv. No 1977:7/3157.1): a body fragment of massive S-shaped vessel with all-over regular fingernail impressions. It was recovered from unseparated Neolithic occupation layer underlying humus, in area 3 quadrat A15 in 1984 (Fig 1). Foodcrust adhered on inner surface was radiocarbon dated 4336–4241 calBC (ARR 15046). The shard was attributed to the Friesack-Boberger group ([24] Abb. 6.7; [25] Abb.6.1).

**#3078 (**Inv. No 1977:7/3078): a rim shard of a funnelbeaker decorated with three irregularly placed angular impressions. The shard was recovered from humus mixed cultural layer of quadrat D5 in 1983 (Fig 1). Charred foodcrust on the internal surface was radiocarbon dated 3628–3376 calBC (ARR 15044) ([24] Abb.6.1; [25] Abb.7.3).

The shards were gently cleaned with a brush to remove loose soil contaminants, wrapped in paper and stored at the Brandenburger Office for Cultural Heritage Preservation. The access for researches may be gained at: Brandenburgisches Landesamt für Denkmalpflege und Archäologisches Landesmuseum (BLDAM), Wünsdorfer Platz 4–5, 15806 Zossen, Deutschland. Proteomics analysis of Friesack samples was carried out in the course of the project “Archäologische Untersuchung des mesolithischen Wohnplatz bei Friesack” of BLDAM under the guidance of Dr.Wetzel and therefore required no further authorization.

### Modern reference samples

Fresh carp roe was purchased at the fish farm Heinz Mueck Fischhandel GmbH, Dresden; fish muscle tissue (*S*. *salar* from aquatic culture, Norway*)* for cooking experiment was obtained from a local vendor in Dresden. For preparation of fish broth, a portion of ca 125g of fish muscle tissue was cooked for 30min in ca 300ml of salty water on low hit in an open pot. Then an aliquot of broth was spin down, supernatant mixed with the SDS-sample buffer and separated on 1D SDS PAGE. A sample of pottery glue (Paraloid^TM^ B-72, Kremer Pigmente GmbH & Co. KG, Aichstetten, Germany) was provided for the analysis by the workshop of Brandenburger Archaeological Museum in Wünsdorf, Germany.

### Radiocarbon dating of #3258

^14^C dating was carried out at the Poznań Radiocarbon Laboratory, Poznań, Poland *(*Poz-80609) leaded by Prof. T. Goslar. The data were analysed with OxCal software v.4.2.4 [37] and calibrated using intCal13 atmospheric curve [38].

### Proteomics analysis

20-35mg of encrusted organic deposits from internal surface of #3251.1, #3157.1 and #3078 shards and from the base of #3258 were scratched off using disposable scalpel and spatula. Each foodcrust was independently sampled twice except for #3251.1 (due to lack of available material). The material was first crashed with a disposable pestle into fine powder in an Eppendorf tube. Then proteins were extracted with 65 mM Tris HCl buffer (pH 6.8) containing 2% sodium dodecylsulfate (SDS) and loaded together with the slurry on a pre-cast 1 mm 12% polyacrylamide gel (BioRad Laboratories, Munich, Germany). After electrophoretic separation the gel was visualized by Coomassie staining, sample gel lane cut into 10 slices each of which was then processed individually. Each gel slab was cut into 1x1mm cubes, washed twice with 50% acetonitrile in 50mM ammonium bicarbonate and dehydrated with neat acetonitrile. Reduction and alkylation steps were not performed to minimize losses of peptides caused by multiple side reactions [39]. Then the gel pieces were rehydrated in 10mM ammonium bicarbonate containing 10% of acetonitrile and 12ng/μl trypsin (modified, sequencing grade, Promega, Mannheim) and their protein content overnight *in-gel* digested at 37°C. The resulting peptide mixtures were extracted twice with exchange of 5% formic acid in water and 100% acetonitrile, the extracts pulled together and dried down. Then the peptides were re-suspended in 20μl of 5% formic acid spiked with GluFib peptide as an internal standard and subjected for LC-MS/MS analysis.

Proteins from modern reference samples and a portion (20mg) of pottery adhesive were extracted, processed and analysed by proteomics on the same way.

Peptides recovered from each gel slab were separately analysed by LC-MS/MS on an Ultimate3000 nano-UPLC system interfaced on-line to a LTQ Orbitrap Velos or Orbitrap HF mass spectrometer (Thermo Fisher Scientific, Bremen, Germany). Sample injection volume was 5μl. The UPLC system was equipped with Acclam PepMap^tm^ 100 75 μm x 2cm trapping column and 75μm x 15cm separating column packed with 3 μm diameter C18 particles (Thermo Fischer Scientific, Bremen). Peptides recovered from each gel slab were separated using 180min linear gradient (solvent A– 0.1% formic acid in water, solvent B– 0.1% formic acid in acetonitrile). Spectra were acquired using top-20 data-dependent acquisition program with 25 s dynamic exclusion time and lock mass option as described in [40]. Spectra were converted into mgf (Mascot Generic Format) format and combined into a single file. Database search was consequently performed by Mascot software v.2.2.04 (Matrix Sciences Ltd, London, UK) against all species, *Actinopterygii* and collagen sequences in NCBI protein database (April 2015, 64057457 entries); reference fish samples were searched against *S*.*salar* or *C*.*carpio* protein sequences in NCBI database (112177 and 118017 entries respectively) under the following settings: precursor mass tolerance 5ppm; fragment mass tolerance 0.5Da or 0.025Da for LTQ Velos and Orbitrap HF respectively; enzyme specificity–trypsin; maximal number of allowed miscleavages–two; variable modifications–methionine and proline oxidized; N-terminal protein acetylation; cysteine propionamide; asparagine and glutamine deamidated. Protein hits were accepted if matched by minimum two unique peptides satisfying the following stringent criteria: peptide sequences contained more than seven amino acid residues, peptide ion scores exceeded the MASCOT homology threshold and was above the value of 30. The results of the database search for fish reference samples were evaluated by Scaffold software (Proteome Software, Portland, v.4.7.5); peptide and protein false discovery rate (FDR) was set at 1% and minimal number of matched peptides at two.

Sample analysis was carried out in compliance with protection guidance for archaeological samples [41]. To avoid cross-contamination with other biological samples during electrophoretic separation a set of electrophoretic equipment was exclusively used for the separation of archaeological samples and void gel was run prior each of them. No molecular weight markers were loaded onto gel to avoid protein horizontal transfer. Disposable pestles were used to homogenize or crash samples; disposable Petri dishes were used for staining, storage and cutting of SDS PAGE gels. Sample preparation was carried out in a laminar flow hood, all solvents were prepared freshly. Ancient samples were not processed or analyzed in one batch with modern references. To avoid carryover during LC-MS-MS runs, three to five blank runs were performed before each analysis using the same gradient program. Blank injections were spiked with peptide calibration mixture (5fmol/μl, Pierce, Rockford, IL) to monitor the instrumental perfomance. Spectra acquired in the last blank run were searched against actual UniProt database without species origin restrictions. Additionally, a list of proteins identified in ten LC-MS/MS runs performed for *in-gel* digests of void gel slabs was used to sort out typical protein contaminants introduced by sample preparation and handling.

To estimate relative deamidation rate of identified glutamine (Q) containing peptides, intensity of peptide peaks was calculated by MaxQuant software (v. 1.6.1.0) [42] using default settings; match-between-runs and LFQ functions were disabled; peptide score threshold was set at 30. Then the peak intensity of deamidated form was normalized to the sum of intensities of both, modified and unmodified forms for each peptide. Peptides including cysteine and miscleaved peptides were excluded. Where specified, deamidation rate was additionally verified by manual peak integration using Xcalibur software (Thermo Fischer Scientific, Bremen, Germany).

Where specified, sequence similarity search for peptides identified by proteomics was performed by MS BLAST program (http://genetics.bwh.harvard.edu/msblast/index.html) [43] against NRDB95 database (v.2016_06); protein BLAST was performed on https://www.ncbi.nlm.nih.gov/ against NCBI protein database.

### Scanning Electron Microscopy (SEM)

Uncoated dry samples were fixed on a metal holder and imaged by SEM on a Magellan 400 FEG-SEM instrument (FEI, Eindhoven, The Netherlands) in immersion mode with accelerating voltages of 3 and 5 kV, beam current 100 pA under variable magnification.

## Results

### Morphological analysis, age and typology of the vessel #1977:7/3258 T

First, it was necessary to define the age and typology of the selected pottery fragments. Attribution and ^14^C radiocarbon dating of ceramics #3251.1, #3157.1 and #3078 were reported previously [24, 25] whereas the pottery #3258 was not analysed. #3258 comprised 12 middle-size to small potshards originating from same vessel which was broken during excavation. The fragments included well preserved lip and basal shards and allowed reliable reconstruction of the pottery profile.

**Fig 2:**
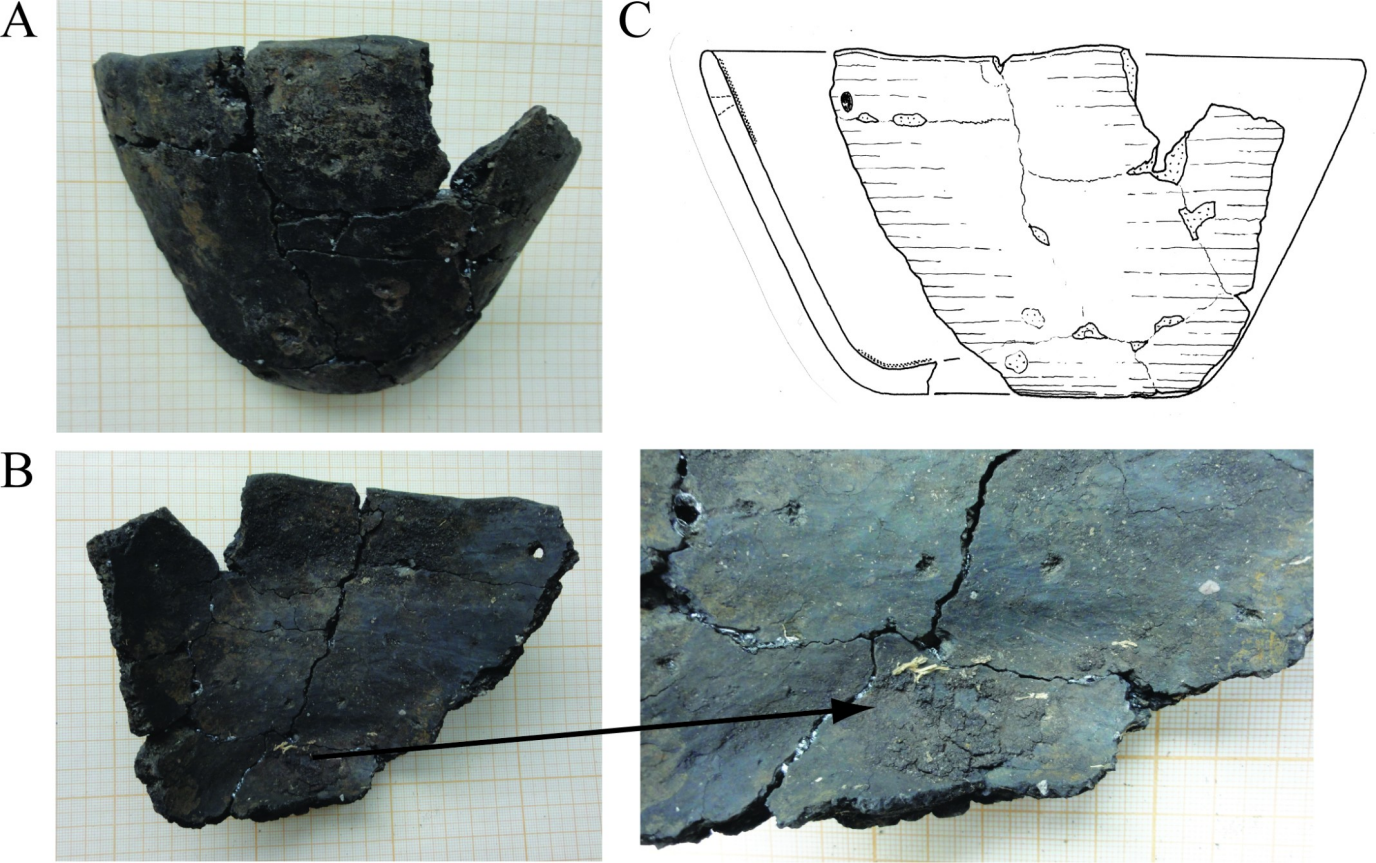
Fragment of Endmesolithic pottery #1977:7/3258 T from Friesack 4. Panel A: side view; Panel B: inner view, secondary conical puncture is seen at the upper right corner. Panel on the right shows basal fragment of the dish with foodcrust. Panel C: Graphical reconstruction of the profile of the bowl #1977:7/3258 T.

The vessel with the height of 10.5cm and 20cm round opening had straight rim and rounded lip, conically ascended wall and rounded flat base with 9.0cm diameter (Fig 2). The pottery was made of medium coarse fired clay tempered with grit and had well smoothed, unglazed, dark brown surface. The body and basal fragment of the vessel are remarkably massive– 0.7cm at the top and 0.6cm at the bottom with 1cm-thick base getting slightly stronger toward the middle. One of the rim shards had secondary conical puncture with 0.5cm outer and 0.3cm inner diameter located ca 1.5cm under the edge of the lip. This presumably mending hole was drilled from outside inwardly after pottery burning (Fig 2). Small amount of dry organic material was adhered as a narrow strip on the inner surface along the rim and as a few millimetre thick dark brown deposit with cracked texture on the base inside of the vessel #3258.

^14^C radiocarbon analysis of the crust from the rim of the #3258 defined a broad time range of 4300 to 4000 calBC (S1 Fig). Considering that freshwater reservoir effect might also significantly alter radiocarbon dating [13] and that its offset is not established for the site of excavation, we also looked at other artefacts recovered in the close proximity to #3258. Two potshards—#3256.1 attributed to the Endmesolithic Friesack-Boberger group [24] (S2 Fig) and #3251.1 assigned to Early Neolith–were found in layers N2 and N5 at the same trench respectively (Fig 1). These age estimates concurred with ^14^C dating of #3258 and positioned the sample in the time span between Endmesolithic and Early Neolith. The combination of unusually massive walls, rounded base and rim of the undecorated coarse conical vessel #3258 did not occur in Neolithic pottery found in the Brandenburg area. Pottery with flat base and conical walls appeared in the region in Rössener culture (site Dyrotz 37 [25, 44]) which is relatively isochronal with Friesack 4 finding, though rounded base, rounded rim and the thickness of walls are not typical for Rössener ceramics. Considering radiocarbon dating, pottery specific characteristics and additionally the fact that the potshards #3258 were found in the lowest occupational layer of the trench, we attributed #3258 to the Endmesolithic pottery of the recently described Friesack-Boberger group [24, 25]. Friesack-Boberger group has certain similarity with Ertebølle ceramics which was replaced by Funnelbeaker pottery at around 4000BC [45]–the time point which also coincides with the #3258 dating.

### Protein composition of foodcrusts from Friesack 4 pottery

Proteomics identified from 70 to 300 proteins of different organismal origin in foodcrusts from Friesack 4 pottery (S1 File). To facilitate the interpretation, proteins considered as a common background were grouped together and remaining protein hits were grouped in four categories according to their organismal origin (Table 1, S1 File): 1. microbial, 2. plant, 3. animals and 4. fish. Next, we calculated deamidation rates in detected glutamine-contained peptides (S1 File). Deamidation of asparagine (N) and particularly of slowly converting glutamine (Q) is an indicator of protein aging and degradation [46, 47]. Pronounced deamidation of these residues was reported for diverse archaeological materials analysed by mass spectrometry [16–20, 48–50]. Deamidation rate of glutamine whose slower turnover is compatible with the time span of archaeological samples was also suggested as an useful marker for identification of outliers and distinguishing contaminants [18]. We calculated deamidation rate of glutamine in reference human keratins and compared it with other proteins identified in archaeological samples.

**Table 1:** Overview of Friesack 4 foodcrusts.

N	Sample name	Archaeological inventory N	^14^C dating		Ceramics typology	Proteomics analysis result (number of identified proteins)^1^		
			BP	calBC		Contemporary proteins^2^		Ancient proteins^2^
						Common background	Environmental contaminants	
1	**#3258**	1977:7/3258 T	5350±30	4300–4050	Friesack-Boberger	Human proteins (32); Hen egg ovalbumin; Bovine milk proteins (1)	Bacteria: *Streptomyces* spp (177), other (7) Fungi (6)	Fish vitellogenins and parvalbumins
2	**#3251.1**	1977:7/3251.1	5042±25	3946–3776	Brzesc Kujawski (?) [24, 25]	Human proteins and multispecies hits (90); Bovine milk proteins (4); Bovine collagen (2)	Bacteria: *Streptomyces* spp (169); other (27) Fungi: *Fusarium oxysporum* (11); other (12) Plants: *Cucurbita maxima*, phloem (2)*;Betula pendula*, pollen (1)	[Collagen, *Suidae* (2)]^3^
3	**#3157.1**	1977:7/3157.1	5419±27	4336–4241	Friesack-Boberger [24, 25]	Human proteins (34); Bovine milk proteins (2)	Bacteria: *Streptomyces* spp (8); other (2); Fungi (13)	
4	**#3078**	1977:7/3078	4705±22	3628–3376	Funnelbeaker [25]	Human proteins (42)*; Picea sitchensis* (2); Bovine milk proteins (2); Bovine collagen (2)	Insect proteins (5) Plants: *Triticeae spp*, seed (5); Fam. *Lamiaceae*; green (1)	

1 –full list of identified proteins is shown in the S1 File.

^2^ –based on deamidation rate of glutamine residues in detected peptides.

^3^ –small number of detected glutamine-containing peptides and overlapping pig and bovine sequences did not allow more specific conclusions.

#### Contemporary proteins in Friesack 4 samples

Common protein background comprised human keratins together with several skin and saliva proteins introduced during sample handling, the majority of them are listed in available predefined contaminants database for proteomics (for example, for MaxQuant software at http://www.coxdocs.org). Conserved proteins whose organismal origin cannot be determined because they are sharing identical matching peptides, e.g. histones or actin, and laboratory standard (hen egg ovalbumin) [51] were also included in this group. Because the fragments of the vessel #3258 T were glued together before sampling the foodcrust, we also analysed a sample of the pottery adhesive and added identified proteins to the background list (S1 File). Interestingly, minute amount of silk protein sericin (gi 112984400, *B*.*mori*) which is used as a moisturizing agent in modern skin and hair cosmetics (up to 20% w/w in some cream formulations) [52] was identified in the glue sample. We then calculated relative deamidation rate in glutamine-containing peptides matched to five most abundant keratins present in each sample (S1 File). We selected only fully tryptic peptide comprising single glutamine residue. Deamidated and unmodified forms of a peptide can be clearly distinguished by LC-MS/MS analysis: they have different m/z and eluted at different retention time (S3 Fig). Altogether, 34 unique keratin peptides comprising single glutamine residue were detected in both native and deamidated forms in all analysed samples (Table 2, S1 File). Average deamidation rate for all these peptides was 0.4% and did not exceed 2.2% which corroborates with estimations for bulk deamidation reported for keratins [18]. Relative deamidation rate in asparagine peptides was at the similar range (2%, calculated for 36 individual sequences).

**Table 2:** Glutamine deamidation rates in fish proteins detected in #3258 and modern reference samples^1^.

Proteins	Number of proteins	Number of peptides containing one glutamine residue^2^	Averaged glutamine deamidation rate, %
Keratins in all samples	5	34	<1
Sample #3258			
Vitellogenin	1	3	96
Parvalbumin	1	1	80
Fresh carp roe			
Vitellogenin^3^	1	3	<1
Parvalbumin^3^	1	1	<1
Proteins in fish broth	12	65	5

^1 –^Raw data are in S1 File

^2^ –Includes all peptides containing one glutamine residue that were detected in Sample #3258; in keratins and fish broth proteins only peptides detected in both native and deamidated forms were considered. Note that for the vast majority of glutamine containing peptides in modern proteins we observed no deamidation.

^3 –^the same peptides as in sample #3258

Bovine milk proteins were detected in all Friesack foodcrusts (Table 1). In total, 26 peptides matching various bovine milk proteins contained glutamine residues. Considering that none of glutamine or asparagine residues in peptides matched to milk proteins was deamidated (S1 File), although they are observed intensively modified in ancient dairies [16], and milk proteins were also detected in control samples (pottery glue and void SDS gels), they were considered contemporary contaminants.

Microbial proteins: the bulk of remaining proteins (except for crust #3078) has microbial origin (Table 1, S1 File) and includes species typical for the natural microorganismal community of a peat bog. Most of identified microbial proteins were matched to *Streptomyces* spp.—a bacteria which is common to humid soils and might be involved in lysis of cellulose [53, 54]. Other detected microbial proteins belong to bacteria inhabiting meromictic freshwater lakes as well as fungal proteins from plant-associated genera *Fusarium* and from plant pathogens occurring in microbiome of peat bog [55]. Though, no *Aspergillus* spp which are typical for destructive microflora on archaeological artefacts [56] were detected. Deamidation rate of asparagine and glutamine residues in microbial proteins was the same as in background keratins.

Plant proteins were identified in crusts #3078 and #3251. They originated from seeds of a plant of *Triticeae* family, green of a plant from *Lamiaceae* family, phloem of squash *Cucurbita maxima* and birch pollen (Table 1, S1 File). Although some of these proteins could be associated with foods, particularly seeds from *Triticeae* plant, deamidation rate of glutamine residues in correspondent peptides was also the same as for keratins. We assumed that these proteins together with a few insect proteins detected in #3078 belong to modern environmental contaminants.

#### Collagens in Friesack 4 foodcrusts

Two core collagens—alpha1(I) and alpha2(I)—matching altogether 25 peptides were identified in foodcrusts #3078 and #3251.1 and, in trace amounts, also in the control sample (pottery adhesive) (Tables 1 and 3). Whereas the majority of collagen-matched peptides was identical to several animal collagens, a few of them in #3078 and control sample were unique for protein entries from *Bovidae* (acc no P02453 and P02465) and in crust #3251.1—from pig (acc no A0A1S7J210 and A0A1S7J1Y9). Notably, asparagine and glutamine residues in collagen peptides were partially deamidated only in crust sample 3251.1. Composition, species specificity and deamidation of detected peptides (Table 3) suggested that foodcrusts #3078 and #3251.1 comprised modern *Bovidae* collagen as a contaminant. In addition, the foodcrust #3251.1 also included minute amount of, likely ancient, pig collagen.

**Table 3:** List of peptides matched to collagens in foodcrusts #3078 and #3251.1.

Peptide sequence^1^	Precursor ion, m/z	Best Mascot peptide ion score(N of matched spectra)^2^		ID of the best scored spectrum	Present in collagens of animal groups^3^	Deamidation state of Q and N ^4^		
		#3078	#3251.1			#3078	#3251.1	Control sample (pottery glue)
Collagen-alpha1(I) chain								
(R)GVVGLPGQR	449.759	**27**(4)	**28**(1)	Ww11_01.02342.02342.2 W15_05.09390.09390.2 glue_06.01952.01952.2	b,s,h,1,2,3,4,5,6	● (-)	● (+/-)	● (-)
(R)GVQGPPGPAGPR	553.292	33(4)	46(4)	Ww11_01.01812.01812.2 W15_05.06740.06740.2 glue_05.01420.01420.2	b,s,h,1,2,3,4,5,6	● (-)	● (+/-)	● (-)
(R)GSAGPPGATGFPGAAGR	730.351	55(4)	77(3)	Ww11_03.02882.02882.2 W15_05.11204.11204.2 glue_06.02345.02345.2	b,s,h,1,2,3,4,5	●	●	●
(R)GQAGVMGFPGPK	589.288	**28**(1)	48(1)	Ww11_03.02751.02751.2 W15_05.10708.10708.2	b,s,h,1,2,3,4,5,6	● (-)	● (-)	
(R)GEPGPAGLPGPPGER	718.345		52(2)	W15_05.11435.11435.2	b,s,h,2,5		●	
(R)GETGPAGPAGP**I**GPVGAR	780.912	34(6)		Ww11_03.05551.05551.2	b,2	●		
(R)GETGPAGPAGP**V**GPVGAR	773.900		**29(1)**	W15_05.14431.14431.2	s,h		●	
(R)GPSGPQGPSGPPGPK	666.831	37(1)		Ww11_03.01617.01617.2	b,s,2,5,6	● (-)		
(R)GPPGPMGPPGLAGPPGESGR	916.935	31(6)		Ww11_03.04710.04710.2	b,s,h,1,2,4,5	●		
(K)GEAGPSGPAGPTGAR	641.313	41(1)		Ww11_03.01481.01481.2	b,4	●		
(R)GFPGLPGPSGEPGK	664.827		33(2)	W15_05.17242.17242.2	b,s,h,1,2,3,4,5,6		●	
(K)GANGAPGIAGAPGFPGAR	793.883		52(4)	W15_05.17008.17008.2	b,s,h,1,2,3,4,5		● (+/-)	
(R)GVPGPPGAVGPAGK	596.820		32(1)	W15_05.08507.08507.2	b,s,h,1,3,5,6		●	
Collagen-alpha2(I) chain								
(R)IGQPGAVGPAGIR	596.84			Glue_06.03455.03455.2	b			● (-)
(R)GPPGESGAAGP**T**GPIGSR	790.887	59(3)		Ww11_01.02629.02629.2	b,h,1,2	●		
(R)GPPGESGAAGP**A**GPIGSR	775.884		34(1)	W15_09.09529.09529.2	s,3		●	
(R)GFPGSPGNIGPAGK	644.322	30(1)		Ww1_03.02841.02841.2	b,1,2,3	● (-)		
(R)GDGGPPGATGFPGAAGR	737.341	52(2)		Ww11_03.02904.02904.2	b,s,1,2,4	●		
(R)GEPGPAGAVGPAGAVGPR	766.895	37(3)		Ww11_03.04284.04284.2	b,2	●		
(R)GLPGVAGSVGEPGPLGIAGPPGAR	1066.058	45(2)		Ww11_01.10429.10429.2	b,s,1,2,4	●		
(R)GIPGP**A**GAAGATGAR	620.326		61(2)	W15_05.09594.09594.2	s,3		●	
(R)GIPGP**V**GAAGATGAR	634.340		34(1)	W15_05.11721.11721.2	b,h,1,2,4,5,6		●	
(R)GIPGEFGLPGPAGPR	727.380		39(1)	W15_05.21986.21986.2	s,6,4		●	
(R)GIPGEFGLPGPAG**A**R	714.368		37(1)	W15_05.21102.21102.2	b,1,2,3,5		●	
(R)TGETGASGPPGFAGEK	739.843		50(1)	W15_05.09791.09791.2	s		●	

^1^—only peptides longer than seven amino acids and with Mascot ion score >30 are included. Species-specific amino acids in homologues peptides sequences are marked in bold. Oxidized proline and methionine residues are underlined.

^2^ –Bold–accepted after manual inspection. includes spectra matched with ion score >15.

^3^—sequences of detected peptide were compared with collagens of animal species (as in NCBI database, Oct. 2017) which inhabited Friesack region in Late Mesolithic and Early Neolith: families *Bovidae* (b), *Suidae* (s); *Caprinae* (1), *Cervidae* (2), *Equidae* (3); order *Carnivora* (4); *C*. *canadensis* (5), *O*.*cuniculus* (6) and human (h).

^4^—detected peptides are indicated with “●”; “-”stays if glutamine or asparagine residue was detected unmodified; “+/-”if both forms, deamidated and unmodified, are detected.

#### Fish proteins in the foodcrust #3258, their properties and species attribution

**Fig 3:**
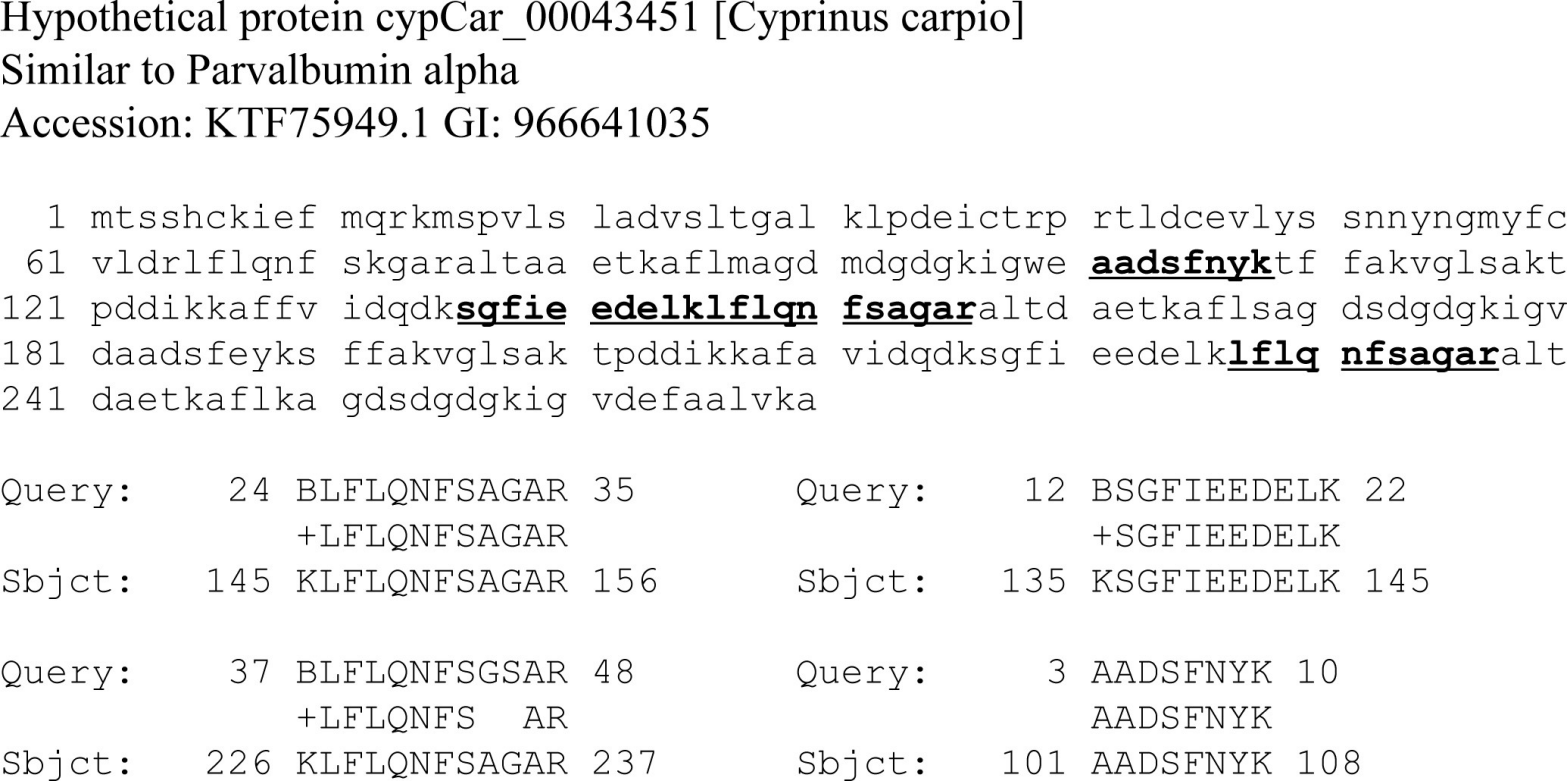
Parvalbumin peptides detected in #3258 basal crust are matched to *C*. *carpio* sequence by MS BLAST. “Query” includes sequences of peptides identified in the sample; “Sbjct”—sequences of corresponding peptides matched to *C*.*carpio* protein (acc no KTF75949.1).

Two fish proteins, vitellogenin and parvalbumin, were matched with seven and three peptides respectively, and were only identified in the fooodcrust from the base shard of the #3258 vessel (Tables 1 and 4). Four out of seven vitellogenin peptides were unique for *Cypriniformes* order including common carp *Cyprinus carpio* and the remaining three were present in multiple vitellogenin sequences from various *Actinopterygii* (Table 4). Detected parvalbumin peptides belong to a conserved part of the sequence and are shared between many fish species. MS BLAST sequence similarity search matched them all together to a single sequence from common carp (*Cypriniformes)* as the top hit (Table 4, Fig 3). The results suggested that the foodcrust #3258 comprised protein material from a *Cypriniformes*. There are no evidence that fish proteins are a part of the sediment at the site: samples 3258 and 3251.1 which were recovered in the striking distance from each other (thin layers 4 and 5 of the test trench, Fig 1) revealed common environmental background largely consisting of Streptomyces spp. whereas fish proteins were identified exclusively in #3258.

**Table 4:** List of peptides matched to fish vitellogenin and parvalbumin in foodcrust #3258*.

Peptide sequence^1^	Precursor ion, m/z	Best Mascot peptide ion score (N of matched spectra^2^)	ID of the best scored spectrum	Gene Identifier of sequences matched by MSBLAST^3^	Organismal order	Relative deamidation of glutamine (%)^4^	
						#3258	Reference sample (fresh carp roe)
**Vitellogenin**							
VQVDAILALR	549.830	54(8)	W2_01.19607.19607.2 ro-03.15419.15419.2	399761434, 4572552, 15778562, 156713467, 124518427, 556825564, 157419156, 284097898, 332712555, 662036448, 831290949	*Cypriniformes*, *Clupeiformes Characiformes*	● (100%)	● (<1%)
LELEVQVGPR	570.817	38(5)	W2_01.11233.11233.2 ro-02.12343.12434.2	399761434, 4572552, 15778562, 742165772, 108863148, 556825564, 157419156, 284097898, 332712555	*Cypriniformes*, *Esociformes*	● (88%)	● (<1%)
AYLAGAAADVLEIGVR	794.939	72(7)	W2_01.13204.13204.2 ro-02.29364.29364.2	399761434, 4572552, 15778562, 124518427, 15778562, 157419156, 284097898, 662036448	*Cypriniformes*	●	●
AEAGVLGEFPAAR	664.339	36(1)	W2_01.07052.07052.2 ro-01.13019.13019.2	399761434, 4572552, 15778562, 156713467, 108863148, 556825564, 284097898, 63100501, 662036448	*Cypriniformes*	●	●
E[I/V]VMLGYGXXXXK5	759.380	50(9)	W2_01.07946.07946.2 ro-02.20727.20727.2	742165772, 1024933123, 662036450, 4572552, 157419156, 399761434, 124518427, 160333705, 1025281290	*Cypriniformes*, *Esociformes*	●	EVVMLGYGSMIAR6
GIINLLQLNVK	613.870	36(3)	W2_01.15246.15246.2 ro-03.22789.22789.2	15778562, 742165772, 5725516, 499021581, 554825686, 86143840, 156530049	*Cypriniformes*, *Esociformes*, *Cichliformes*	● (100%)^8^	GILNILQLNLK^6^^,^^8^ (<1%)
LPIIVTTYAK	559.85	37(2)	W2_01.12404.12404.2 ro-07.09971.09971.2	156713467, 691424740, 1020581238	*Cypriniformes*	●	LPITVTTYAK^6^
**Parvalbumin**							
AADSFNYK	458.214	36(2)	W2_0_02576.02576.2	966641035^7^	*Various Actinopterygii (including Cypriniformes)*	●	*n/d*
SGFIEEDELK	583.783	33(3)	W2_0_08991.08991.2 ro-10.06024.06024.2	966641035^7^	*Various Actinopterygii (including Cypriniformes)*	●	●
LFLQNFS[AG/GS]AR5	613.31	35(2)	W2_01.09714.09714.2 ro-10.08132.08132.2	966641035^7^	*Various Actinopterygii (including Cypriniformes)*	● (80%)^8^	LFLQNFSAGAR (0%)^8^

*—data are combined from two analyses

^1^—only peptides longer than seven amino acids and Mascot ion score above 30 are included. Underlined amino acids were detected modified

^2^ –includes spectra matched with ion score >15.

^c^–statistically significant hits matching identical peptide sequence or its homologues resulted from single nucleotide substitution (as for example valine to isoleucine replacement).

^4^ –“●”–stays for peptides detected in the sample; “n/d.”–no peptide detected

^5 –^matches to several overlapping fragmentation spectra acquired from precursors with different mass

^6^ –matched to homologue sequence

^7^ –accession number of the best MS BLAST protein hit matching all detected peptides

^8^ –peptide GIINLLQLNVK was detected in three forms in #3258 (15% Q, 69% QN and 16% QNN-deamidated) and two forms in fresh roe (Q and QN-deamidated, both <1%). Peptide LFLQNFSAGAR was detected in #3258 as N- and QN-deamidated (20% and 80% respectively) and unmodified in fresh roe.

Vitellogenins are the precursors of yolk proteins which make up the bulk of egg in fish [57]. They are synthetized in female liver as preproproteins and then delivered by bloodstream to ovaries where they are processed and accumulated. Ovaries are most enriched with vitellogenin compared to other organs of the fish body: its content exceeds 2.5pmol/μg of total protein [58]. The amount of vitellogenins in fish liver and in blood is 500 folds lower than in ovaries [58] and their expression in other tissues, e.g. skin or muscles, is below 10% of that in liver [59]. Vitellogenins were not detected by proteomics in fresh carp liver and muscles [60, 61]. Taking together, vitellogenins could be considered as marker protein specific for fish eggs. No major proteins from tissues, in which vitellogenins could be also present (e.g. blood hemoglobin or muscle myosin), were detected in crust #3258. Therefore, we assumed that one of the ingredients of the meal in #3258 was fish caviar or whole roe. To further test this we analysed fresh carp roe sac (unseparated eggs and skin) (S1 File). As anticipated, vitellogenins constituted the bulk of proteins in roe. More than 40% of the total of 65 000 MS/MS spectra matched vitellogenins sequences.

Parvalbumins are small proteins with remarkable chemical and thermal stability and high solubility in water that are ubiquitously expressed within the fish body [62]. Two parvalbumins were also detected in trace amounts in fresh carp roe (S1 File). Thus, one of plausible explanations of why parvalbumin was present in the foodcrust #3258 might be that it originated from roe sac together with vitellogenins. Parvalbumins are enriched in white (non-fatty) muscles of fishes [63] from which they can be readily extracted by boiling [64]. Thus, the presence of parvalbumins might also indicate that the composition of ancient meal #3258 included fish broth. To test this hypothesis we prepared fish broth and analysed it by proteomics. Indeed, four proteins of parvalbumin family were found among 339 proteins detected in the broth and one of them (acc no ACH71041.1, matched with 300 spectra) was among top ten most abundant proteins in the sample (S1 File).

**Fig 4:**
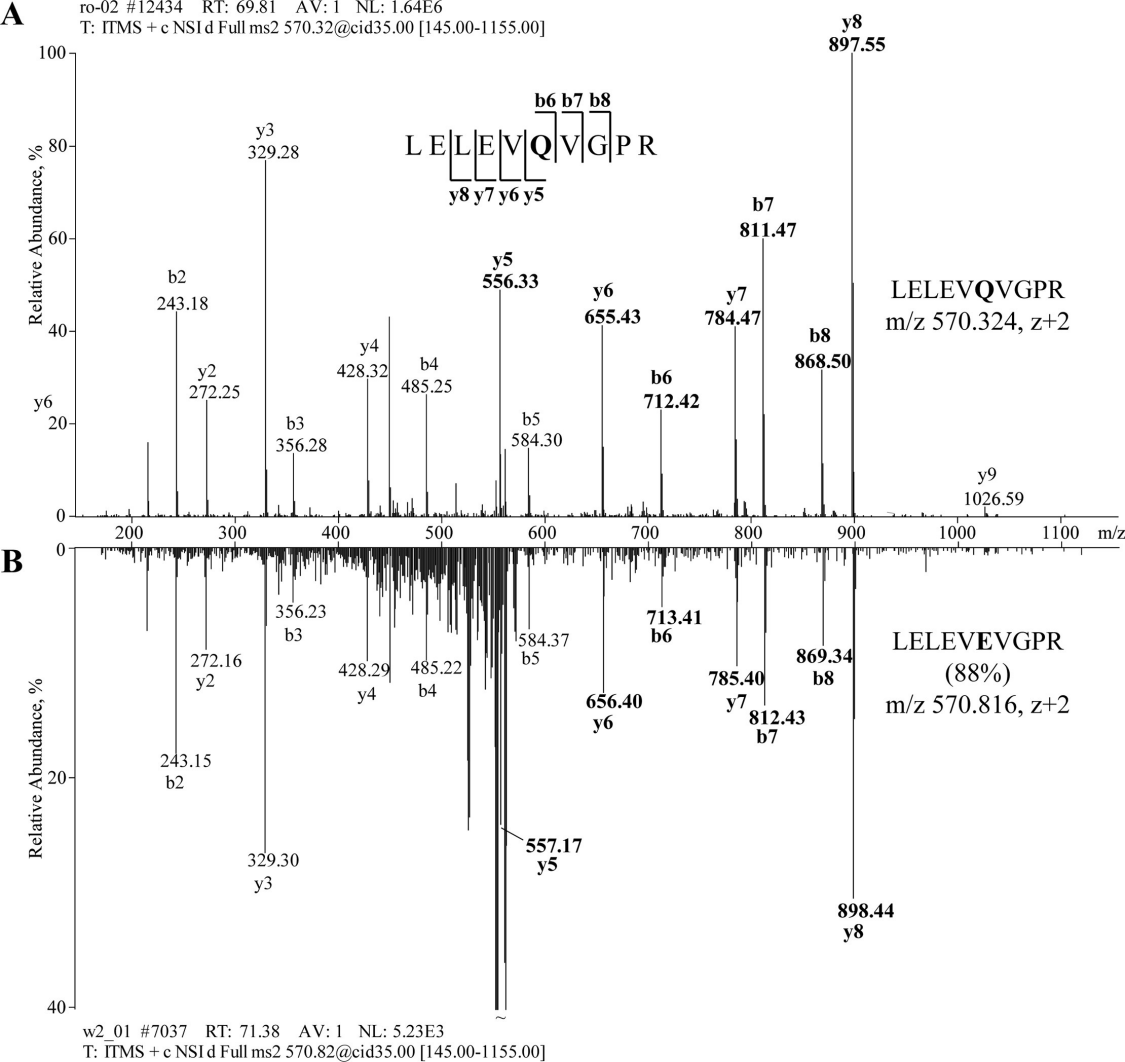
Pronounced deamidation of the glutamine residue (Q) in fish vitellogenin from foodcrust #3258. Upper panel: Fragmentation spectrum of vitellogenin peptide LELEV**Q**VGPR (m/z 570.32, z +2) identified in fresh carp roe. In foodcrust #3258, 88% of glutamine residue in this peptide was converted into glutamic acid (E); mirrored image of fragmentation spectrum of peptide LELEV**E**VGPR detected in #3258 is shown at the lower panel. Masses of fragment ions including Q or E at the position six are shown in bold.

Remarkably, in contrast to other proteins identified in Friesack samples, all glutamine residues in peptides matched to vitellogenin and parvalbumin in #3258 were detected strongly deamidated (Fig 4, Table 4). Deamidation rate of the same peptides in fresh roe did not exceed background level of 1% indicating that their deamidation does not naturally occur in living fish (Table 2, S1 File).

Could sample #3258 be occasionally contaminated with thermally processed fish during handling? To exclude this possibility we calculated glutamine deamidation rate in 12 most abundant proteins identified in the fish broth (Table 2, S1 File). These proteins matched 65 tryptic peptides comprising one glutamine residue which were detected partially deamidated, 34 of them belonged to soluble fraction of collagens. Each protein was presented by more than one glutamine-contained peptide. Average deamidation rate was ca 5% and, importantly, it did not significantly vary between proteins. An exception was a single collagen peptide QGPGGPVGER that was fully deamidated. However, the deamidation rates in other peptides belonging to the same protein did not differ from the average; moreover the peptide was not detected in oxoproline-containing form and therefore it seems likely that here the quantitative estimates are biased by incomplete detection of its relevant molecular forms. Taking together, pronounced glutamine deamidation of fish proteins in residue #3258 cannot be solely explained by food cooking and we concluded that both vitellogenin and parvalbumin proteins were ancient.

### Electron microscopy of foodcrusts from pottery #3258

**Fig 5:**
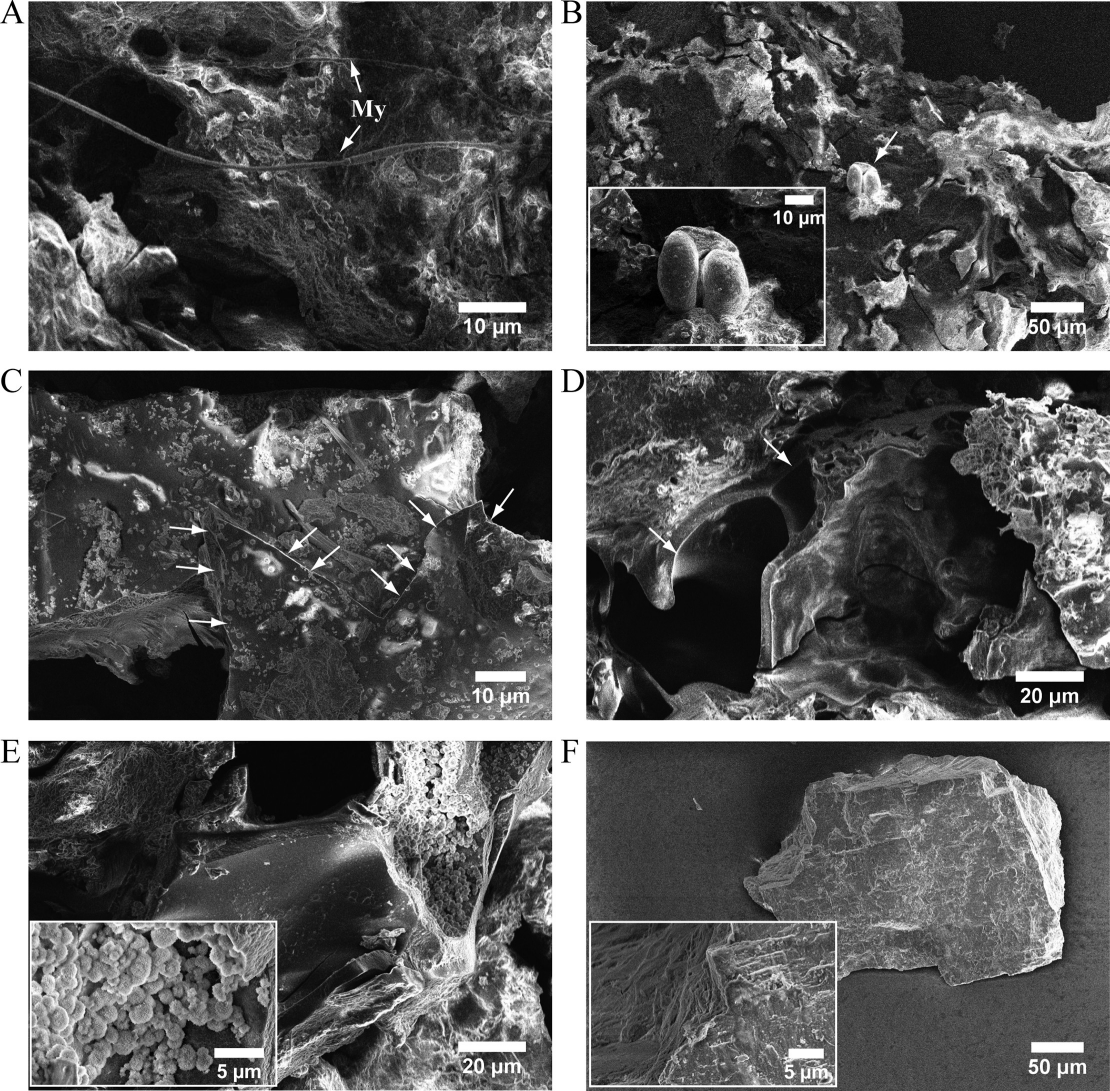
Electron microscopy of rim and basal foodcrusts from #3258 vessel. Panels A-E: Images of complex rim crust with fungal micelles (My), incrusted bisaccate conifer pollen (panel B) and Streptomyces-type bacterial spores (panel E) on the surface. Damaged microstructures of plant phloem and xylem, leaves surface cuticle, frazzles of thin membranes and fluffy bulk material are designated with arrows on panels C and D, and also well recognizable on panel E. Panel F: A piece of basal crust consisting of dense homogeneous material.

Electron microscopy of the rim and basal crusts from #3258 pottery (Fig 5) did not detect fish microremains although microscopic fish bones and scales were observed in ancient foodcrusts [65–67]. It corroborates with our assumption that the whole fish was not cooked in this pottery.

Rim and basal crusts had different texture and density. The rim deposit comprised plant microremains. Vascular cuticles and microstructures of damaged xylem and phloem of leaves and stems—50–70μk tubes ensheathed by ca 2μk smooth wall—are seen on electron microscopy images (Fig 5). At some positions, the surface of the rim residue was covered with a dense layer of what might be Streptomyces-like bacterial spores with “fluffy” ornamentation, and ca 1μm salt microcrystals. Two typical conifer bisaccate pollens were incrusted into rim deposit. In contrast, the dark brown basal crust had dense and homogeneous texture with a few light-yellowish grains of sand (Fig 5).

## Discussion

### Evidence of Endmesolithic pottery production in the region between Elbe and Oder

Coarse ceramic fragments of conical bowl #3258 and S-shaped vessel #3157 assigned to the Friesack-Boberger group is the oldest pottery found at the Friesack 4. Their morphology differs from other ceramics recovered at the site which are presented by region typical middle and late Neolithic cultures [24, 25]. Similar examples of Endmesolithic vessels which morphology was typologically foreign to the Mesolithic Ertebølle and the Early Neolithic Funnelbeaker pottery dominated at the area were reported earlier at the Boberg site southeast of Hamburg [68–71], and opened a question about ceramic production by Endmesolithic groups in Northern Europe. Based on typology and stylistic details it was also hypothesized that these pottery might be associated with other known cultures [72, 73] or considered as an evidence of adoption and flow of technologies resulted from interactions with early Neolithic settlers [45, 69]. Endmesolithic ceramics of the recently described Friesack-Boberger group recovered at Friesack 4 and Rhinow 30 sites [24, 25] comprise fragments of coarse pottery with certain similarity to Ertebølle and pottery from Boberg (S2 Fig). Inclusion of the well preserved shards #3258 in the group supports the notion that ceramics were manufactured by Endmesolithic hunter-gatherers far south in Northern European inland, and contributes to understanding of Neolithization process in the region between Elbe and Oder. Whether the Friesack-Boberger group was developed under influence of early Neolithic cultures in Havelland reminds an open question. Although there are evidences for elder pottery in the vicinity south and north from Friesack (Linear, Stroke and Rössener ceramics [25, 74, 75]) no links to these groups has been found in Friesack 4 so far.

Shape, undecorated massive walls, flat base, prominent carbonized residue on the internal surface and rim crust assumed that pottery #3258 was used for cooking or stewing, set on open fire or embers. The bowl has relatively small volume and was unlikely utilized for preparation of seasonal scaled stocks or family meal but could be used for cooking of selected foods. The ware certainly belonged to a valuable household utensils: secondary puncture on the wall indicates that when the bowl got a crack it was first carefully mended and later, once probably became unrepairable, finally littered.

### Characteristics of Friesack 4 meals

Organismal origin of mixed collagens in foodcrust #3251.1 could not be unequivocally attributed by proteomics. *Suinae* collagen identified in foodcrust #3251.1 could indicate that pork with bones, sinews or skin was cooked in this pottery. It correlates well with Neolithic zooarchaeological artefacts from Friesack 4 which include boar bones [33], and corroborates the notion that this site was used as a hunting station. However, small number of detected glutamine-containing peptides and overlapping pig and bovine sequences (Table 3) did not allow more specific conclusions.

How carp roe was processed in the small bowl #3258? Fish roe is an ingredient of many traditional recipes. It is considered delicacy and can be consumed grilled, fried, marinated, baked, smoked, dried, cured and also boiled in broth [76]. Proteomics did not find evidences of fish fermentation, although this food processing practice was exercised by costal settlers of the Northern Europe already in early Mesolithic [77]. No microorganisms which might have been associated with fish fermentation, as for example *Tetragenococcus halophilus*, [78–81] were identified in the foodcrust. Charred deposit in the interior of the bowl indicated that the food was most likely thermally processed. Size and shape of the pottery, charred crust and identification of parvalbumin suggested that delicate carp roe might be cooked in a small volume of water or fish broth, for example by poaching on embers. Charred plant remains observed on electron microscopy images encrusted in the organic deposit from the rim of the bowl (Fig 5) assume that the pot was probably capped with leaves.

Local geological prerequisites [82] and historical reports describing natural crystallization of salt in the direct proximity of the site [83] indicated that Friesack foods might be also salted. Fried-clay moulds for salt extraction (or briquetage) from nearby Halle a.d.S. dated on the 3^rd^ millennium BC indicated that saltern was practised in Neolith in the region [84–86].

### Fish meal #3258 and exploitation of aquatic resources at the Friesack 4 site

Fishing was an important part of subsistence strategy at Mesolithic Friesack. This notion is supported by a variety of recovered artefacts: bast knotted nets, birch-bark net-floats, fragments of dug-out canoe,—and rich ichtioarchaeological data [28, 30]. However, no fishing or fish processing tools elucidating role of aquatic resources in transition and early Neolith were recovered at the site. Fish could be caught even with very simple traps during spring flooding and spawning season and the lucky finding of carp roe meal in pottery #3258 evidenced that fishing practice continued at the Friesack site also in earlier chronological period. 3% of totally 21 thousand faunal remains recovered from the Neolithic occupational layer [33] are bones from fishes also including carp. It affirms that *Cyprinoformis* belonged to site-specific aquatic species at Neolithic Friesack and corroborates with our findings.

In this work, proteomics revealed details of prehistorical culinary recipes by direct analysis of charred foodcrust. This work also highlighted the impact of sample heterogeneity and possible contamination with environmental proteins on data interpretation. Being recovered from drained and cultivated peat bog, Friesack samples revealed high environmental background represented by remains of agricultural plants and abundant soil bacteria *Streptomyces spp*.–one of a few known bacterial genera which accumulates fats (triacylglycerols) [87, 88]. Whereas proteomics distinguished between ancient and contemporary materials, common lipid- or fatty acid-based method as for example CSIA (compound specific stable isotope analysis) might be affected by presence of modern bacterial lipids in the sample.

## Supporting information

S1 Fig ^14^C dating of the rim crust from pottery #3258. (TIF)

S2 Fig Early ceramics from Friesack 4 and Rhinow 30 tentatively attributed to Friesack-Boberger group. All ceramics except for #1 and #2 were excavated at Friesack 4; the #1 and #2 fragments–at Rhinow 30 site located 20km to the west from Friesack. Pottery fragments with inventory numbers “If” are stored in the Berlin State Museums, the remaining—in the BLDAM (Wünsdorf, Germany). Apart of ceramics shown on Fig S2, to the Friesack-Boberger group very likely might be also attributed ceramic excavated at the Friesack site by M.Schneider [25, 26]. Unfortunately these objects were lost during the Second World War. 14C-dating is shown where available. **2845/3 (Inv. No 1977:7 2845/3):** 1x body shard, coarse fired clay tempered with grit/quartz, outside light brown, inside brown-grey, slip-glaze, smooth and crackly inside, 1,0cm thick, from the lower part of a vessel with probably ca.12cm diameter. **2846.4 (Inv. No 1977:7 2846.4):** 1x rim and 3x body shards of a bowl, rounded lip, ochre-brown, outside roughened probably with stalks, inside smooth, medium coarse fired clay tempered with grit, 0.5cm thick. **2949.1** and **2 (Inv. No 1977:7 2949.1 and 1977:7 2949.2):** 1x rim and 1x body shards of jar or bowl with ca 30cm diameter, rounded lip, well smoothed surface, brown to dark-brown, medium coarse fired clay tempered with grit, 0.7cm thick. 14C dating rim shard: 4546–4366 calBC (external crust) (Poz-84049), 4857–4709 calBC (internal crust) (Poz-84050). **3038.3** (Inv. No 1977:7: 3038.3): 1x body shard, probably from a conical lower part of a vessel with ca 10cm diameter, outside grey-brown, inside and edge light brown/ochre, smooth, coarse fired clay tempered with coarse grit, 0.65–0.7cm thick, fine triangular point impressions on the outer surface. **3085.1 (Inv. No 1977:7 3085.1):** rim shard of a flat bowl with 26cm diameter, square-edged slightly rounded uneven lip, grey-brown with dark spots, medium coarse fired clay temperate with grit, 0.6–0.7cm thick. ^14^C dating: 3778-3648calBC (Poz-84051). **3127.6 (Inv. No 1977:7 3127.6):** 2x body shards of a round bowl with ca 28cm diameter, 0.6cm thick, fingertip impressions with unclear orientation, inside/outside light grey-brown. **3157.1 (Inv. No 1977:7 3157.1):** 2x body shard from shoulder of a S-shaped vessel with ca 30cm diameter, all-around impressions of vertically oriented fingertips arranged in horizontal lanes, middle coarse fired clay, tempered with grit, friable, inner black, outside brown. 14C dating: 4336-4241calBC (ARR 15048). Early Neolith? Brzesc Kujawski? **3171/1 (Inv. No 1977:7 3171/1)**: 3x body shards of a round-bodied vessel with neck/shoulder, ca 30-34cm diameter, 0.7–1.0cm thick, middle coarse fired clay tempered with grit, outer surface is structured with press- and point-impressions, porous and rough internal surface, outside grey-brown, inside black-grey. **3251.1 (Inv. No 1977:7 3251.1):** 1x body shard, shoulder of a vessel with ca 28cm diameter, 0.5–0.6cm thick, middle coarse fired clay tempered with grit, three closely placed drop-shaped impressions, smooth inner surface, grey-brown to dark-brow. 14C dating 3870+/-60 calBC. Early Neolith? Brzesc Kujawski? **3256.1 (Inv. No 1977:7 3256.1):** 1x body shard, vertically orientated fingertips/fingernails impressions arranged in horizontal lanes, coarse fired clay tempered with grit. **3258:** description in the Result section. **If 11710(4):** 1x body shard of a lower part of a vessel with ca 22-25cm diameter, regular 0.6cm drop-shaped tip-over impressions arranged in rows, 1.5cm between impressions, 1cm between rows, ochre grey-brown, smoothly roughed, middle coarse fired clay tempered with grit, 1.0cm thick. **If 11711(9):** 1xbody shard, all-around fingertips prints, temper pulled-up with nails, grey-brown, middle coarse fired clay tempered with grit, smooth inside, diameter ca 32-34cm, 0.7–0.9cm thick. **#1** (Inv.no. 1995:375/2/126): Rim, body and base shards of a vessel with S-shaped profile and conical base, decorated with 4-5mm round imprints, fired clay tempered with sand and grit, outside burnt; 24-28cm, height 29-30cm; walls 0.9cm, 1.6cm at the base. **#2** (Inv.no. 1995:375/2-169,171) 2x body shards of a S-shaped vessel, decorated with irregular imprints, fired clay tempered with sand and grit; 26cm diameter, wall 0.5–0.6cm.
(TIF)

S3 Fig Example of the calculation of deamidation rate of glutamine-containing peptides. Glutamine residue in peptide LELEVQVGPR matching multiple *Cyprinoformis* vitelogenin sequenecs was partially deamidated in #3258. Left hand side panel; XIC of the peptide in the first analysis, right panel–the second analysis. Both forms of the peptide with precursor m/z 570.325 and 570.817 for unmodified and deamidated forms, respectively, are eluted at different retention time. Timing of MS/MS events triggered at the eluted peaks are indicated as vertical bars. The formula for calculating deamidation rates is shown below the panels; A stays for peak areas at XIC traces.
(TIF)

S1 File List of proteins and peptides identified in archaeological and reference samples by proteomics. (XLSX)
